# Underwater Techniques in Gastrointestinal Endoscopy: Diving into the Depths

**DOI:** 10.3390/cancers16203535

**Published:** 2024-10-19

**Authors:** Sandro Sferrazza, Giulio Calabrese, Roberta Maselli, Rui Morais, Antonio Facciorusso, Georgios Mavrogenis, Roberto Di Mitri, Alessandro Repici, Marcello Maida

**Affiliations:** 1 Gastroenterology and Endoscopy Unit, ARNAS Civico Di Cristina Benfratelli Hospital, 90127 Palermo, Italy; sandro.sferrazza@arnascivico.it (S.S.); giulio.calabrese@unina.it (G.C.); roberto.dimitri@arnascivico.it (R.D.M.); 2 Digestive Endoscopy Unit, Humanitas Clinical and Research Hospital, Rozzano, 20089 Milan, Italy; roberta.maselli@hunimed.eu (R.M.); alessandro.repici@hunimed.eu (A.R.); 3 Department of Biomedical Sciences, Humanitas University, Pieve Emanuele, 20072 Milan, Italy; 4 Gastroenterology Unit, Unidade Local de Saúde São João, 4200-319 Porto, Portugal; u011791@chsj.min-saude.pt; 5 Section of Gastroenterology, Department of Surgical and Medical Sciences, University of Foggia, 71122 Foggia, Italy; antonio.facciorusso@unifg.it; 6 Unit of Hybrid Interventional Endoscopy, Department of Gastroenterology, Mediterraneo Hospital, 16675 Athens, Greece; mavrogenis@gmail.com; 7 Department of Medicine and Surgery, University of Enna “Kore”, 94100 Enna, Italy; 8 Gastroenterology Unit, Umberto I Hospital, 94100 Enna, Italy

**Keywords:** underwater, resection, EMR, ESD, POEM

## Abstract

The endoscopic resection of gastrointestinal tract lesions embraces different types of techniques, ranging from conventional polypectomy/endoscopic mucosal resection (EMR) to the field of third-space endoscopy, including endoscopic submucosal dissection (ESD), full-thickness resection and peroral endoscopic myotomy (POEM). Parallelly, the advent of underwater techniques has served as an add-on for both basic and advanced procedures, since its first report in 2012. We aimed to provide a comprehensive update on the state of the art about the feasibility of underwater basic and advanced techniques for GI endoscopy. Underwater EMR (U-EMR) has proved effective and safe in treating > 10 mm sessile or flat or all-size recurrent colonic lesions. Conversely, although data show good effectiveness and safety for <10 mm lesions, it is preferred when high-grade dysplasia is suspected, favouring cold snare polypectomy for all other cases. Moreover, promising data are emerging regarding the feasibility of U-ESD for difficult-to-resect colonic lesions. U-EMR represents a standard of care for treating < 25 mm superficial non-ampullary duodenal epithelial tumours. Data regarding oesophageal, gastric and ampullary lesions remains limited to small cohorts. Finally, using water immersion for POEM has shown a reduction in procedure time compared to the CO_2_ insufflation technique for vessel coagulation, albeit in a single-centre experience. Based on these results, U-EMR has become a standard for treating intermediate-size colonic and non-ampullary duodenal lesions, as highlighted also in the European Society of Gastrointestinal Endoscopy guidelines. Promising results have been shown in third-space endoscopy studies, even though further prospective studies are awaited to standardise the technique for both ESD and POEM.

## Simple Summary

The advent of underwater endoscopic resection techniques has served as an add-on for both basic and advanced procedures, since its first report in 2012 till its inclusion into the European Society of Gastrointestinal Endoscopy guidelines. Hence, we aimed to perform a comprehensive update on the state of the art about the feasibility of underwater basic and advanced techniques for GI endoscopy. Underwater endoscopic mucosal resection represents a standard for treating intermediate-size colonic and non-ampullary duodenal lesions. Promising results have been shown in third-space endoscopy studies, even though further prospective studies are awaited to standardise the technique for both endoscopic submucosal dissection and peroral endoscopic myotomy.

## 1. Introduction

The endoscopic resection of gastrointestinal (GI) tract lesions, including polyps and early tumours, is considered an essential skill for all endoscopists [1]. This procedure has been demonstrated to reduce the rate of surgery and mortality in populations at risk of developing cancer [2,3]. A wide range of resection techniques have been described and adopted, showing different degrees of difficulty mainly based on the site and type of resection [1]. Polypectomy is considered a basic technique for resection of <20 mm polyps: it can be performed with (“lift polypectomy”) or without submucosal injection and either with hot or cold snare placement (cold snare polypectomy—CSP) [1]. Conventional endoscopic mucosal resection (EMR) consists of the submucosal injection of different solutions and is usually performed for lesions > 20 mm in diameter [1]. In 2012, Binmoeller [4] first reported the possibility of using water as a lumen-filling agent to favour lesion lifting and provide a stable and effective resection without performing submucosal injection. During the following years, the so-called “underwater revolution” [5] spread, and several studies have proven the efficacy and safety of this technique [6,7,8,9,10], leading to its inclusion in the updated version of the European Society of Gastrointestinal Endoscopy (ESGE) guidelines on colonic polypectomy [1].

At the same time, the advent of third-space endoscopy has revolutionised the field of endoscopic resection and treatments. New techniques, such as endoscopic submucosal dissection (ESD) and full-thickness resection (FTR), have pushed the borders of endoscopic resection yield, giving the possibility of removing early tumours in one piece (*en bloc*) and resulting in curative treatment, thus avoiding surgery in selected cases [11,12,13]. Based on the principle of “third space access”, peroral endoscopic myotomy (POEM) and septotomy (POES) have been developed as therapeutic approaches for achalasia and Zenker’s diverticulum [14,15]. Despite the high therapeutic potential of the latter techniques, acquiring the necessary skills to perform them is lengthy, limiting their widespread adoption [16]. Additional factors such as the involved organ, disease location and intrinsic lesion characteristics (morphology, size and presence of fibrosis) play a critical role in influencing the complexity of the procedure [17,18]. In this context, water or saline immersion has shown the potential to represent a game-changer in helping endoscopists gain stability and effective lifting. Consequently, the amount of literature published on this specific topic has progressively grown.

We conducted a narrative review of the underwater basic and advanced interventional techniques in the GI tract, in an attempt to provide an updated overview of this field.

## 2. Principles of Underwater Resection Technique

As demonstrated by endosonographic studies, water immersion causes mucosal and submucosal layers to act like gastric folds and lift well, contrary to the muscle layer, which remains deep. That phenomenon happens due to the antigravity effect of submucosal fat tissue [19]. This enables snare grasping to be effective and safe without requiring submucosal injection. Moreover, water immersion stabilises the lumen and guarantees better endoscopic visibility under challenging locations, like the right colon or second part of the duodenum [20,21].

As a technique, underwater EMR (U-EMR) is performed after the lumen is completely filled with water or saline (200–1000 mL usually). Margins of resection are traditionally marked either with an argon plasma coagulation (APC) probe or a snare tip, especially for large lesions. Afterwards, a snare is placed around the marking dots, and current is applied for resection. The most common electro-surgical unit (ESU) settings comprise autocut effect 5 or dry-cut effect 5 [22].

## 3. Organ-Specific Approaches to Underwater Resection Techniques

### 3.1. Oesophagus

#### 3.1.1. Underwater Endoscopic Mucosal Resection and Submucosal Dissection

According to the updated ESGE guidelines, oesophageal EMR is strongly recommended as a standard of care for ≤20 mm Barrett’s oesophagus lesions with a low probability of submucosal invasion (Paris type 0-lla, 0-lIb) and for larger or multifocal benign (dysplastic) lesions [23]. Conversely, ESD is considered the treatment of choice for all-size superficial squamous cell carcinoma lesions, given the higher 5-year survival rates in patients who received that treatment [24]. Doumbe-Mandengue reported oesophageal U-EMR [25] as a successful treatment of a 15 mm polyp. The floating effect of water provided effective visualisation of the tumour border and snaring of the lesion. The pathological report resulted in an adenocarcinoma with free resected margins. Regarding additional techniques, Deng [26] performed an oesophageal U-hybrid ESD for a 30 mm lesion. Given the relatively small lesion size, snare resection was preferred over submucosal dissection after the circumferential incision to reduce possible adverse events. After a 20 min procedure, a 42 × 30 mm specimen was sent for histopathological evaluation, revealing a squamous cell carcinoma confined to the lamina propria with free lateral and vertical margins.

#### 3.1.2. Underwater Peroral Endoscopic Myotomy and Septotomy

POEM briefly consists of an oesophageal myotomy performed after gaining access to the submucosal space [15]. According to the Society of American Gastrointestinal and Endoscopic Surgeons (SAGES) [27] guidelines, POEM is the preferred treatment for Type I and Type II achalasia. Compared to surgery, it has lower rates of adverse events [27]; however, the occurrence of pneumoperitoneum consequent to CO_2_ insufflation is not negligible [28]. Consequently, a saline immersion technique for POEM (U-POEM) has been adopted to overcome this risk. In 2016, Binmoeller [29] reported two successful cases of Type II achalasia treated with U-POEM, mentioning no end-tidal CO_2_ or airway pressure increase at the end of the procedure. At the same time, Hallit [30] performed three cases of U-POEM with saline, describing a minimal pneumomediastinum in one case. Interestingly, the coagulation setting was set on spray coagulation effect 4. More recently, Capogreco et al. [31] reported a series of U-POEMs focusing on saline role in vessel pre-sealing during the submucosal dissection phase through a swift coagulation (effect 3) compared to a standard soft coagulation approach. Specifically, they found a lower rate of use of coagulation forceps and tool exchange in the U-POEM group vs. standard of care, resulting in a reduced procedure time (*p* = 0.014) and comparable outcomes.

An adaptation of POEM is POES, first described by Repici et al. [32] in 2020, which consists in a Zenker’s septum myotomy after creating two paired submucosal tunnels. The latter is considered an ideal technique for short Zenker’s diverticulum (<20 mm), which may not allow stable access for traditional myotomy or submucosal tunnelling, like in Z-POEM [33]. The same group recently pushed its limits on this concept of accessibility of small Zanker’s diverticulum, performing a saline immersion POES (U-POES) [34]. The use of saline immersion to gain effective access to the submucosal tunnels of the diverticulum allowed scope stability, which was not possible with CO_2_ insufflation.

An overview of all the underwater oesophageal interventional techniques is summarised in Table 1.

### 3.2. Stomach

According to the updated version of the ESGE guidelines [23], all gastric lesions suspected to be dysplastic should be treated to achieve *en bloc* resection, with the treatment of choice being ESD. EMR can be considered an alternative when the lesion size is less than 10 mm and the operator feels confident about achieving complete resection in one piece. Water or saline immersion can help the operator optimise lesion lifting for EMR and ESD and reduce procedure time. The first underwater resection for a non-pedunculated gastric lesion was reported by Iwagami [35] in 2019. The author reported using U-EMR to resect a remaining early gastric cancer after ESD interruption for perforation. Water immersion could allow for an optimal base definition and help an *en bloc* resection in a short time. Since then, few series have been published on this kind of resection (Table 2) [36,37].

However, some retrospective data have been collected and published in recent years. In 2022, Yamamoto [39] reported 36 cases of gastric U-EMR on different size lesions, reporting a 100% *en bloc* and a 72.4% R0 resection rates. Even though inferior to ESD, in that case-series U-EMR showed good feasibility in a location with greater curvature (traditionally considered as challenging in ESD) with an R0 resection rate of 82% and a short procedure time (mean 6.3 min). Moreover, the technique showed a 100% R0 rate in case of lesions < 15 mm, in contrast with previous data on gastric conventional EMR [47]. Shimamoto also confirmed the feasibility of U-EMR on greater curvatures [42], finding comparable outcomes between U and conventional EMR in terms of safety and procedure duration but lower rates of R0 resection for the first one, despite not reaching statistical significance.

Interestingly, Kim [43] recently conducted a retrospective study on 81 gastric lesions (mean size = 10.1 ± 2.8 mm): U-EMR had 100% rates of *en bloc* resection, with 93.4% being R0. However, in five cases, ESD was performed due to cascade stomach conformation, which was not ideal for effective water filling, thus hampering U-EMR. The authors concluded that U-EMR could be a valid alternative to ESD for lesions < 10 mm, especially in frail patients, but also more convenient than conventional EMR, carrying the advantages of a shorter time and no need for an injection. Conversely, the literature regarding gastric U-ESD is somehow poor, with reported cases limited to the water pressure method [45]. In this regard, we report a recent case by Muramatsu [46], who performed a U-ESD with a conventional technique on an upside-down stomach due to a prolapse into the thoracic cavity caused by a huge hiatal herniation. In this case, water/gel immersion was crucial even to reach the lesion stably, thanks to the guarantee of a reduced intraluminal pressure compared to CO_2_. Hence, the operator completed the resection with free lateral and vertical margins in the histopathologic report.

### 3.3. Duodenum

#### 3.3.1. Underwater Resections of Superficial Non-Ampullary Duodenal Epithelial Tumours

EMR is the preferred choice for the endoscopic treatment of superficial non-ampullary duodenal epithelial tumours (SNADET) [48]. Despite its increasing incidence [49], SNADET can present some challenges. First, an optical diagnosis lacks clear accuracy in histologic prediction; thus, there is a need to perform pre-resection biopsies, which can induce fibrosis. Secondly, the poor scope manoeuvrability, especially in the second portion of the duodenum, and the reduced endoscopic view induced by lesion lifting easily occur during resection. Hence, conventional EMR can become difficult for both expert and non-expert endoscopists. Based on the experience developed for colonic lesions, Binmoeller published a first case series of duodenal U-EMR in 2013 [50]. Among the 12 lesions selected, the technical success rate was 91.7%, with one lesion resected on a two-session procedure and one refusing to undergo the second resection, preferring surgery.

This sample’s rate of adverse events was not negligible, as shown in Table 3. However, the median lesion size and circumferential involvement were 35 mm (IQR = 20–150) and more than one-half, respectively, in 25% of cases. Those lesions were difficult-to-resect and to date they would be scheduled for ESD, in order to obtain a more accurate specimen and better control adverse events.

Conversely, U-EMR has been adopted for small (<25 mm) SNADET and is now considered an alternative technique for improving outcomes, as stated in the latest ESGE guidelines [48]. This assumption comes from many studies published during the last few years. The first structured prospective study on ≤20 mm lesions was conducted by Yamasaki [51]: among the 31 lesions included, the rate of *en bloc* resections was 87%, with an R0 of 61%. However, the complete resection rate was high, with a recurrence rate of 3%. The same group recently coordinated a multicentre randomised controlled trial (RCT) called the D-UEMR Study, including 21 Japanese institutions [7]. The number of lesions included in the analysis was 166, with a mean size of 9.8 ± 4.7 mm and showing outcomes comparable to the previous experience with an *en bloc* rate of 89.8%, an R0 rate of 66.9% and a recurrence rate of 3.6%. The *en bloc* resection rate significantly decreased between lesions sized more or less than 10 mm (78.5% vs. 100%; *p* < 0.001), without decreases in nonrecurrence rate (97.4% vs. 97.5%). Previous studies reported a relatively high recurrence rate: in a retrospective study by Iwagami [52], 4.6% of patients undergoing follow-up showed a lesion recurrence, which was subsequently treated with either U-EMR or cold U-EMR. Regarding the direct comparison with conventional EMR, literature data show a relative advantage for U-EMR. Despite being retrospective studies, Kiguchi, Toya and Tanaka [53,54,55] reported comparable outcomes between those techniques (see Table 3), with a higher feasibility of the underwater technique. Notably, in a recent retrospective study conducted by Morais et al. [56], piecemeal U-EMR was performed for >20 mm lesions, showing no differences in terms of technical success compared with conventional EMR and a reduced use for margin coagulation.

Regarding adverse events, clip closure generally ensures low rates of post-procedural bleeding and delayed perforation. Of note is that aspiration pneumonia can occur due to the high quantity of solutions administered into the bowel lumen; hence, many authors advise orotracheal intubation [51,57].

Two case reports concerning U-ESD are available in the literature. In 2018, Nagata [58] reported a U-ESD of a 25 mm superior duodenal angle: gravity did not favour an ideal distribution of liquids in that specific location, which impaired a feasible dissection. The author took advantage of this condition by switching from CO_2_ to saline immersion, thus better recognising wall layers and effectively completing the resection. More recently, Santos-Antunes [59] presented a case of U-ESD of a large adenoma located at the distal second duodenal portion: water immersion effectively allowed for a safe trimming, given the lower tendency of the duodenal mucosa to contract after circumferential incision, as it usually happens in other organs.

**Table 3:** Overview of duodenal underwater rection techniques.

Author	Design	Year	Country	Technique	Device	ESU Setting	Lesion Size	Lesion/Case Number	Lesion Site	*En Bloc*/Technical Success	R0	Recurrence	AEs	Comparative
Binmoeller [50]	Prospective	2013	USA	U-EMR	Duckbill 15 mm snare	Dry Cut (E 5)	>20 mm	12	2nd duodenum: 12 (100%)	91.7% *	-	0% ^	Bleeding: 3 (25) Other (stricture): 1 (8.3)	-
Yamasaki [51]	Prospective	2018	Japan	U-EMR	Round stiff 10–15 mm snare	Endocut Q (E 3, I4, L 2)	<20 mm	31	Bulb 4 (13) 2nd portion 26 (83) 3rd portion 1 (3)	87%	61%	3%	Other (aspiration pneumonia): 1 (3)	-
Kiguchi [53]	Retrospective	2020	Japan	U-EMR	Round stiff 10–20 mm snare	-	<20 mm	90 (PP)	Proximal: 19 (18%) Distal: 85 (82%)	87%	67%		Bleeding: 2 (2)	Conventional EMR: *en bloc*: 96%, R0: 80%
Iwagami [52]	Retrospective	2020	Japan	U-EMR	Round stiff 10, 15, 25 mm	Forced pre-coag. (2) Endocut Q (E 3, I 4, L 2)	All sizes	162	Bulb: 21 (13) 2nd portion: 132 (81) 3rd portion: 9 (6)	68%	46% Unclear: 50%	7/157 underwent follow-up	Bleeding: 3 (1.8) Perforations: 1 (0.6)	-
Toya [54]	Retrospective	2020	USA	U-EMR	Round stiff snare	Endocut Q (E 3, I 4, L 2) Forced coag. (2)	<20 mm	17	2nd portion: 17 (100)	100%	88.2%	-	0	Conventional EMR: *en bloc*: 100%, R0: 95.2%
Furukawa [6]	Retrospective	2021	Japan	U-EMR	Different sizes	Endocut Q (E 3, I 4, L 2)	<20 mm	28	Bulb: 6 (21.4) 2nd portion: 21 (75.0) 3rd portion: 1 (3.6)	96.4%	71.4%	-	0	Conventional EMR: *en bloc*: 72.2%, R0: 50%
Yamasaki [7]	Multicentre prospective	2022	Japan	U-EMR	10–20 mm snare	Endocut	<20 mm	166	Bulb: 10 (6.0) 2nd portion, preampulla: 71 (42.8) 2nd portion, postampulla: 80 (48.2) 3rd portion: 5 (3.0)	89.8%	66.9%	3.6%	Bleeding: 6 (3.6)	-
Miyazaki [57]	Single-centre RCT	2023	Japan	U-EMR	Round stiff 10–13 mm snare	Endocut Q (E 1)	<12 mm	64	Bulb: 7 (10.9) 2nd portion: 57 (89.1)	92.2%	70.3%	0%	Bleeding: 6 (9.4) Other (aspiration pneumonia): 2 (3.1)	CSP: *en bloc*: 95.4%, R0: 61.5%, recurrence: 1.5%
Morais [56]	Retrospective	2024	Europe	U-EMR	Braided or monofilament snare	-	12.0–30.0 mm	89	Bulb: 8 (9) 2nd portion: 80 (89.9) 3rd portion: 1 (1.1)	97.8%	-	9.7%	Bleeding: 10 (11.2) Perforation: 2 (2.2)	Conventional EMR: technical success: 94.5
Tanaka [55]	Retrospective	2024	Japan	U-EMR	Round stiff 10–15 mm snare	-	<20 mm	96	Bulb: 2 (2) Prox. 2nd portion: 51 (53) Distal 2nd portion: 40 (42) 3rd portion: 3 (3)	94%	68%	-	Bleeding: 1 (1.0)	Conventional ERM: *en bloc*: 91%, R0: 56%.
Nagata [60]	Case report	2018	Japan	U-ESD	Waterjet-not-assisted hook tip knife	-	25 mm	1	Superior duodenal angle	100%	100%	-	0	
Santos-Antunes [59]	Case report	2020	Portugal	U-ESD	-	-	60 mm	1	2nd portion	100%	100%	-		
Granata [61]	Case report	2014	Italy	U-papillectomy	-	Dry Cut (E 5)	20 mm	1	Papilla of Vater	100%	100%	-	0	
Yamazaki [62]	Case report	2020	Japan	U-papillectomy	Round stiff 15 mm snare	-	-	1	Papilla of Vater	100%	100%	-	0	
Mori [63]	Case report	2020	Japan	U-papillectomy	-	-	-	1	Papilla of Vater	100%	100%	-	0	

Abbreviations: ESU, electrosurgical unit; U, underwater; U-EMR, underwater endoscopic mucosal resection; U-FTR, underwater full-thickness resection; RCT, randomized controlled trial; CSP, cold snare polypectomy; * two circumferential resections needed two U-EMR sessions; one of the patients preferred to undergo surgery. ^ 0% of those who completed the treatment.

#### 3.3.2. Papilla of Vater: Underwater Papillectomy

Underwater papillectomy encompasses an area that has not been extensively studied to date. It is not possible to state how effective and safe this technique is, given that only a few sporadic studies are available in the literature. However, it is reasonable to speculate that the same concept could be valid for papillary lesions as it is for SNADETs. The first case of U-papillectomy was reported in 2014 [61]. Since then, Yamazaki and Mori reported their experience [62,63]. Yamazaki [62] showed an effective U-papillectomy in a Roun-en-Y patient: in that specific situation, resection was attempted and performed with a short-type double-balloon endoscope. The use of water to reach the papilla could allow for better scope manoeuvrability and may have helped during the resection, guaranteeing more stability. On the other hand, the study by Mori [63] demonstrated that U-papillectomy could help allow successful treatment of residual/recurrent lesions with effective tissue lifting and grasping, as already shown for other bowel districts.

### 3.4. Colon

#### 3.4.1. Underwater EMR for Flat or Sessile Colorectal Lesions

U-EMR is now considered a treatment option for >20 mm colonic sessile or flat adenomatous colonic or sessile serrated lesions with signs of dysplasia without suspicion of submucosal invasion [1]. Binmoeller first described this technique for large colonic lesions [4,22,64] and then applied it as a concept to other locations, as described in the above parts of this manuscript. The innovation provided by this study was represented by the feasibility of the technique and the possibility to also effectively and safely resect non-lifting lesions, where invasiveness was previously excluded. Since then, the technique has spread, and many authors have introduced it in daily practice for colonic lesions, given the emerging literature data on its efficacy (Table 4).

U-EMR has been adopted as hot or cold and for lesions with the widest size range, such as <10 mm, 10–20 mm or >20 mm. Regarding lesions <10 mm, data are available from four different studies [68,72,73,87]. Zhang [72] specifically designed a multicentre RCT on 71 4–9 mm colorectal lesions, achieving a 94.4% *en bloc* rate compared to 91.5% of cold snare polypectomy (CSP), which is the reference standard [1]. Comparable outcomes were also reported by Yen [73] and Cadoni [87]. Even though it has proved effective and safe, performing U-EMR requires a hot snare and ESU; consequently, it can be time-consuming and more expensive compared to a CSP with dedicated snares. Given the high level and established evidence in favour of CSP for <10 mm lesions, it is reasonable to state that U-EMR may not add any advantage for this kind of lesion if there is no suspicion of high-grade dysplasia. Conversely, when endoscopic appearance suggests features of high-grade dysplasia, U-EMR could have a therapeutic role, being less time-consuming and expensive than conventional (submucosal injection) EMR, as stated by Takeuchi et al. [5].

For lesions between 10 and 20 mm, the *en bloc* resection rate of U-EMR tends to be higher than that of conventional EMR. Some studies have been specifically designed on this lesion size. In 2019, Yamashina [70] conducted a comparative RCT at five Japanese institutions, comparing 108 U-EMRs vs. 102 conventional EMRs on intermediate-size lesions: the R0 and *en bloc* resection rates were significantly higher in the U-group compared to the conventional group, with rates of 69% vs. 50% (*p* = 0.011) and 89% vs. 75% (*p* = 0.007), respectively; no significant differences were observed in terms of adverse events. More recently, Kim [79] published a prospective study carried out on 47 10–20 mm size lesions with a modified version of U-EMR, showing a 97.9% *en bloc* resection rate and an 80.9% rate of R0. In this series, the resection was divided into two phases: first, the lesion was inspected and snared under CO_2_, and then, after the grasping phase was concluded, the water immersion with the following resection was performed. According to the authors, this method could guarantee a better lesion inspection and a clear definition of margins; however, safety concerns must be considered, given the relatively high rate of muscle entrapment for intermediate-size lesions (19.1%). Other prospective data are extractable from older studies, including all-size lesions [66,67] and showing *en bloc* resection rates of U-EMR ranging between 82.9 and 100%. Regarding retrospective data, Chaves conducted a propensity score-matched study [69]: in the subgroup analysis based on the polyp size 10–19 mm and including 74 conventional and 74 U-EMRs, he found comparable outcomes in terms of *en bloc* resection rates [71 (96.0%) vs. 72 (97.3%)] with shorter procedure times for U-EMRs (7.1 min vs. 6.1 min). Therefore, U-EMR shows globally high efficacy and safety outcomes for intermediate-size lesions with shorter procedure time compared to conventional EMR.

Regarding large (>20 mm) lesions, Table 4 provides a full overview of U-EMR outcomes. A first prospective study was conducted in 2015 by Binmoeller [22]: among 50 large colorectal lesions, the *en bloc* resection rate was 55% with an R0 rate of 46% and 5% recurrence, showing similar data to those from conventional EMR studies. In a comparative review and meta-analysis [21], including studies directly comparing U- and conventional EMR, both techniques performed similarly for large lesions in terms of *en bloc* resection rate (OR = 0.8; 95% Cis = 0.3–2.1; *p* = 0.75), R0 resection (OR = 3.1; 95% Cis = 0.74–12.6; *p* = 0.14), with lower rates of polyp recurrence for U-EMR (OR = 0.3; 95% Cis = 0.1–0.8; *p* = 0.01). Certainly, *en bloc* resection through EMR can be a target for 20–25 mm lesions, while for lesions larger than 25 mm, ESD should be the preferred technique to achieve it, as highlighted in the comparative study by Okimoto [80] and Inoue [75]. Hence, the results in terms of *en bloc* and R0 resection rates from RCTs tend to be lower for studies including >20 mm compared to 10–20 mm lesions. Nagl [8] included 81 U and 76 conventional EMRs on 20–40 mm lesions, finding *en bloc* resection and R0 rates of 33.3% vs. 18.4% (*p* = 0.045) and 32.1% vs. 15.8% (*p* = 0.025), respectively. He did not find differences in terms of recurrence between the two groups (*p* = 0.253), but he did for 30–40 mm lesions (*p* = 0.03) in favour of U-EMR. Conversely, a subgroup analysis from an RCT conducted by Lenz [82] demonstrated zero versus five cases of recurrence in lesions >20 mm treated with U-EMR and conventional EMR, respectively, with a statistically significant difference (*p* = 0.04). In an attempt to increase *en bloc* resection for >20 mm lesions, Okada [77] published in 2022 a study of U-EMR with progressive polyp contraction: it consisted of the lesion grasping at the far site without closing the snare too tight, progressively reopening it and grasping more tissue with the far portion being hooked and giving a snaring mark as a guide. In this manner, the author increased their *en bloc* resection rate to 91%: the results of this case series seemed promising despite the small sample size (11 lesions). Given that *en bloc* and R0 rates cannot represent optimal outcomes for EMR of large lesions, the recurrence rate tends to be lower in U-EMR compared to conventional EMR. However, RCTs are expected to evaluate this aspect more precisely.

Furthermore, U-EMR has also proven effective for lesions at the appendiceal orifice. In 2016, Binmoeller [64] reported his prospective experience on 27 lesions involving appendiceal orifices with a median size of 15 mm: the *en bloc* resection rate was 59%, the procedure was judged as successful in 89% of cases, and surgery was indicated in the remaining 11%. The recurrence rate was 7.4%, and all patients received a new endoscopic treatment. Similarly, Uchima [78] described cap suction U-EMR for appendiceal orifice and non-lifting lesions: this technique seemed promising in achieving neglectable rates of recurrence (1.6%; 1 out of 64 underwent follow-up).

#### 3.4.2. Underwater ESD and Third-Space Technique for Colorectal Lesions

Over the last few years, the underwater technique has also become widely used in colorectal ESD. Despite the lack of indication for performing a specific technique and the low level of evidence, water immersion has been proven to be as effective and safe as other methods, such as conventional, pocket-creation, or traction-assisted methods. The use of saline solution instead of water increases the cutting performance of the knife compared to the sole water immersion [88]. Nagata [60] first described U-ESD in 2017: he retrospectively reported a 100% *en bloc* rate on 26 lesions with a median procedure time of 60 min (IQR = 45–111) and median speed of dissection of 10.4 mm^2^/min (IQR = 6.4–12.2). U-ESD is expected to reduce procedure time, providing a more effective trimming due to the lesion buoyancy. Moreover, U-ESD also proved effective for lesions presenting with moderate-to-severe submucosal fibrosis. Among the 40 lesions successfully resected by Yoshii [83], with an 87.5% R0 rate, 23 (57.5%) had moderate-to-severe fibrosis. More recently, Cecinato [84] reported a retrospective study on 59 “difficult” cases (either presenting with fibrosis, difficult scope position, unfavourable knife angle) treated with either U- or hybrid ESD. Among those cases, 100% of lesions treated with U-ESD were removed *en bloc*, achieving no recurrence, while only 59.5% of hybrid ESD were completed *en bloc* with a 2.7% recurrence rate. Regarding complications, no bleeding or perforation occurred in U-ESD, while 10.8% of hybrid ESD cases experienced it. Moreover, according to a study conducted by Koyama [85], U-ESD showed a significantly lower incidence of post-polypectomy electrocoagulation syndrome compared with conventional ESD (0% vs. 11.1%; *p* = 0.03) with significantly higher dissection speed (6.9 mm^2^/min vs. 10.9 mm^2^/min; *p* < 0.001).

Finally, water immersion can also be applied to FTR. In the case recently presented by Shigeta [86], water immersion made colonic intubation easier with the FTR device mounted on the scope tip, which can raise technical challenges due to its size. Furthermore, water was also a game changer for severe fibrosis of the lesion, which was the residual of a previously complicated ESD and not easy to grasp by snare for U-EMR.

## 4. Discussion

During the last decade, underwater techniques have represented an innovation for GI tract resection, especially for colorectal lesions. Literature data have grown substantially since the first publication on this topic, leading to its affirmation as a standard of care in different situations [5]. In an attempt to provide an updated state of the art of the technique, we conducted the present comprehensive review.

According to the data above, U-EMR is an effective and safe treatment for resectioning >10 mm sessile or flat colonic lesions. Indeed, when the lesion size is greater than 20–25 mm, ESD is the preferred technique, at least for achieving an *en bloc* resected specimen. Even though it has proved effective and safe [89], U-EMR should be attempted for <10 mm lesions only when high-grade dysplasia is suspected. A different discussion is warranted for recurrences, which can benefit from underwater treatment even when small in size. Immersion facilitates lifting the lesion, which might otherwise be insufficiently elevated if injected [90].

Similarly to colonic lesions, U-EMR is also considered an established treatment for small (<25 mm) SNADETs: the quality of evidence has grown over the last years [7], and the comparison with conventional or cold EMR shows advantages for the underwater technique [6,54,57]. For lesions >25 mm, ESD with or without saline immersion or laparoscopic endoscopic co-operative surgery (LECS) can be considered the preferred treatment, especially when the whole circumference is involved and the risk of lymph node metastases is low [48,91].

On the contrary, U-EMR has limited applicability to gastric lesions: ESD is indeed the gold standard treatment for nearly all size lesions. Hence, U-EMR has only been applied to <10 mm lesions in specific settings where ESD was not feasible for technical difficulties (Table 2).

Finally, the data regarding oesophageal lesions and papillectomies remain sporadic and limited to case reports or small cohorts (Table 1 and Table 3).

The current trend in using underwater techniques for first- and second-level resections suggests that saline immersion may serve not only as a rescue method but also as a primary approach for the entire gastrointestinal tract that has already been integrated into practice, especially for the lower GI tract. Immersion stabilises the position of the endoscope, enhances mucosal visualisation and performs a “physical” lifting involving both mucosal and submucosal layers, thereby ensuring a safe resection relative to the muscular plane. For the upper GI tract, it is essential to consider the need for oro-tracheal intubation of the patient to minimise the risk of aspiration pneumonia. Therefore, this technique should be considered primarily for lesions with additional challenges, such as fibrosis or unstable positioning. Moreover, the use of saline (0.9%) instead of sterile water for EMR is primarily desirable to avoid water intoxication, especially in the upper GI tract, due to the high volumes required [88].

Underwater can be a total game changer in third-space endoscopy. Emerging data have demonstrated the benefits and support of saline during POEM or ESD. The integration of methods can overcome challenges during advanced resections (depending on fibrosis, scope instability and lesion morphology). Water immersion can be used in conjunction with traction or pocket-creation methods, facilitating ESD by leveraging the buoyancy effect, which aids in trimming; the hydrating effect, which reduces the need for submucosal injection; and the magnification effect, which enhances the operator’s ability to recognise layers during the dissection phase. The 1.3 refractive index, provided by the immersion, enhances the visibility and permits a safer and faster dissection phase [84,92]. However, some specifications are required. According to the ESGE Technical Review on ESD [93], 0.9% saline should be preferred over water due to better current conduction and reduced osmotic effect, compared to sterile water, which can determine tissue friability. However, the current dispersion, especially for the coagulation effect, is not overcome just by using saline instead of water, and specific ESU power settings have not yet been established for ESD. Authors report different types of coagulation modes, varying from swift effect 3 to forced or spray effect 3, as effective. As can be assumed from Table 1, Table 2, Table 3 and Table 4, heterogeneity remains high in terms of ESU settings, and data lack generalizability due to their restriction to specific last-generation ESUs.

Further studies are indeed awaited in this regard. On the contrary, Capogreco [31] has turned the U-current dispersion into an advantage during tunnelling in POEM: he reports benefits from a swift coagulation effect 3 for underwater vessel pre-sealing, reducing the need for coagulation forceps and reducing the procedure time and device switches, without increasing adverse events. One other challenge of U-ESD is finally represented by the bubble creation from the ESU-generated heath: Sasaki tried to solve this problem [94] by synchronising the water irrigation pump with the ESU activation, thus reducing bubble formation effectively with immediate waterjet.

Finally, according to data from small case series [95], using specific viscous gel solutions could ameliorate visibility compared to water immersion, especially when there is abundant mucus or bleeding occurs, thanks to its three-dimensional composition made of xanthan gum, locust bean gum, concentrated glycerine and purified water; additionally, it could theoretically reduce the risk of aspiration pneumonia.

## 5. Conclusions

**Figure 1:**
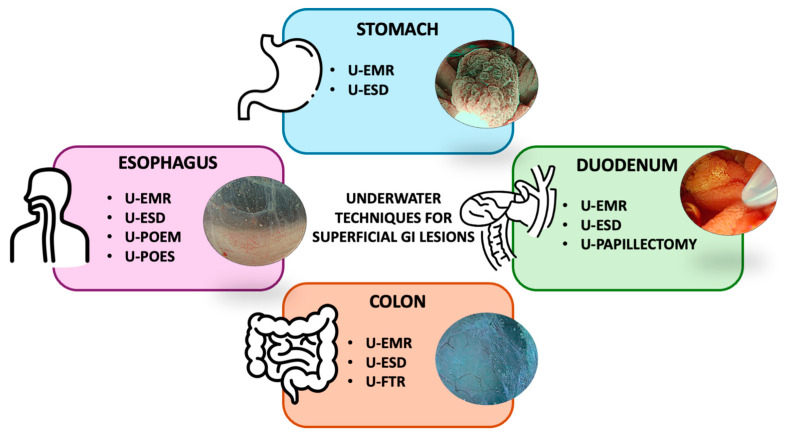
Overview of the principal underwater resection techniques for superficial gastrointestinal (GI) tract lesions. Abbreviations: U, underwater; EMR, endoscopic mucosal resection; ESD, endoscopic submucosal dissection; POEM, peroral endoscopic myotomy; POES, peroral endoscopic septotomy; FTR, full-thickness resection.

In conclusion, underwater techniques have substantially integrated into daily clinical practice as a standard of care treatment for some types of endoscopic resections and are advancing to improve procedure feasibility in third-space endoscopy (Figure 1). Further studies are awaited to find the best settings and understand outcomes to advance underwater-based techniques further.

## Figures and Tables

**Table 1:** Overview of oesophageal underwater endoscopic techniques.

Author	Design	Year	Country	Technique	Device	ESU Setting	Lesion Size/Disease	Lesion/Case Number	Lesion Site	*En Bloc*/Technical Success	R0	Recurrence	AEs	Comparative
Doumbe-Mandengue [25]	Case report	2022	France	U-EMR	15 mm snare	-	20 mm	1	GEJ	100%	100%	-	0	-
Deng [26]	Case report	2023	China	U-Hybrid ESD	Waterjet-not-assisted knife Round 30 mm snare	Endocut Q (E 3, I 4, L 2) Swift coag. (3)	40 mm	1	Oesophagus	100%	100%	-	0	-
Binmoeller [29]	Case report	2016	USA	U-POEM	Waterjet system-assisted knife	-	Type II achalasia	2	Oesophagus	100%	-	-	0	-
Hallit [30]	Case report	2020	France	U-POEM	Waterjet-not-assisted knife	Tunnel and myotomy: spray coag. (4) endocut (E 1/3–3)	Type II achalasia	3	Oesophagus	100%	-	-	1 (minimal pneumomediastinum)	-
Capogreco [31]	Case series	2024	Italy	PUC-POEM	Waterjet system-assisted knife	Swift coag. (3)	-	21	Oesophagus	-	-	-	0	
Maselli [34]	Case report	2024	Italy	U-POES	Waterjet-not-assisted knife	-	Short–septum (20 mm) Zenker diverticulum	1	Proximal oesophagus	100%	-	-	0	-

Abbreviations: ESU, electrosurgical unit; U-EMR, underwater endoscopic mucosal resection; ESD, endoscopic submucosal dissection; GEJ, gastroesophageal junction; U-POEM, underwater peroral endoscopic myotomy; PUC-POEM, prophylactic underwater coagulation peroral endoscopic myotomy; U-POES, underwater peroral endoscopic septotomy.

**Table 2:** Overview of gastric underwater rection techniques.

Author	Design	Year	Country	Technique	Device	ESU Setting	Lesion Size	Lesion Number	Lesion Site	*En Bloc*	R0	Recurrence	AEs	Comparative
Kono [38]	Case report	2018	Japan	U-EMR	-	-	Pedunculated lesion	1	Corpus	100%	100%	-	0	-
Iwagami [35]	Case report	2019	Japan	U-EMR	Round stiff 25 mm snare	Endocut Q (E 3) Forced coag. (E 2)	15 mm	1	Pylorus	100%	100%	-	0	-
Uemura [36]	Case report	2019	Japan	U-EMR	30 mm snare	-	Pedunculated lesion	1	Pylorus	100%	100%	0	0	-
Kim [37]	Case series	2020	Republic of Korea	U-EMR	Crescent-type snare	Endocut Q (E 2, I 5, L 3)	<15 mm	4	Pylorus	100%	100%	-	0	-
Yamamoto [39]	Retrospective	2022	Japan	U-EMR	10–33 mm snare	Endocut Q (E 3) Forced coag. (2)	10 mm (2–50)	36	Upper: 11 (30.6) Middle: 16 (44.4) Lower: 5 (13.9)	100%	72.4%	0	0	-
Tanabe [40]	Case report	2022	Japan	U-EMR	10 mm snare	Endocut Q	5 mm scar	1	Antrum	100%	100%	-	0	-
Deng [41]	Case report	2023	China	U-EMR	-	Endocut Q (E 3, I 4, L 2)	10 mm	1	Antrum	100%	100%	-	0	
Shimamoto [42]	Retrospective	2023	Japan	U-EMR	Round stiff 10–20 mm snare	Endocut Q (E 3, I 4, L 2) Soft coag. (4)	<20 mm	25	-	88%	56%	0	0	Conventional-EMR: *en bloc*: 92%, R0 75%, recurrence: 16.7%.
Kim [43]	Retrospective	2024	Republic of Korea	U-EMR	Hexagonal 15–20 mm snare	Endocut Q (level 2, L 3)	10.1 ± 2.8	76	Cardias: 4 (5.3) Body: 15 (19.7) Antrum: 46 (60.5) Pylorus: 11 (14.5)	100%	93.4%	2.6%	Bleeding: 28 (36.8) Other: 5 (6.6)	Rescue ESD: *en bloc*: 100%, R0: 100%, recurrence: 0
Okubo [44]	Case report	2024	Japan	U-EMR	-	-	25 mm	1	Fundus	100%	100%	-	0	-
Miyazaki [45]	Case report	2022	Japan	U-ESD	Waterjet-not-assisted knife	Dry cut (E 2.5); Swift coag. (E 3.5)	15 mm	1	Lesser curvature	100%	100%	-	0	-
Muramatsu [46]	Case report	2024	Japan	U-ESD	Waterjet-not-assisted knife	-	10 mm	1	Upside-down stomach	100%	100%	0	0	

Abbreviations: ESU, electrosurgical unit; U-EMR, underwater endoscopic mucosal resection; EMR, endoscopic mucosal resection; ESD, endoscopic submucosal dissection.

**Table 4:** Overview of colonic underwater rection techniques.

Author	Design	Year	Country	Technique	Device	ESU Setting	Lesion Size	Lesion Number	Lesion Site	*En Bloc*	R0	Recurrence	AEs	Comparative
Binmoller [22]	Prospective	2015	USA	U-EMR	Stiff braided 33 mm snare	Autocut (E 5)	>20 mm	50	Right C: 38 (76) Left C: 12 (24) R: 3 (6)	55%	46%	5%	Bleeding: 1 (2%)	-
Uedo [65]	Retrospective	2015	Sweden	U-EMR	Stiff rounded 33 mm snares	Endocut (E 2) Forced coag. (2)	>15 mm	11	C: 7 (63.6) AC: 1 (9.1) TC: 2 (18.2) R: 1 (9.1)	55.5%	64%	-	0	-
Binmoeller [64]	Prospective	2016	USA	U-EMR	15–25 stiff snares	Dry cut (E 5)	15 (8–50)	27	Appendiceal orifice	59%	-	2/21 underwent follow-up	Post-polypectomy coagulation syndrome: 2 (7%)	-
Amato [66]	Prospective	2016	Italy	U-EMR	15–32 mm stiff rounded snares	Endocut (E 3)	>10 mm	25	Right C: 18 (72%) Left C: 4 (16) R: 3 (12)	76%	76%	-	0	-
Cadoni [67]	Retrospective	2018	Italy	U-EMR	Polyfilament duckbill or oval snares Monofilament snares	Dry cut (E 5) or endocut Q (E 3) Forced coag. (E 3)	Any size 108 (55.4) flat or sessile lesions 87 (44.6) pedunculated lesions	195	Right C: 38 (19.5) TC: 29 (14.9) Left C or R: 128 (65.6)	87.7%	97.6%, just for sessile or flat lesions	-	Bleeding: 16 (8.2%)	6–9 mm: CSP >10 mm: conventional EMR *En bloc*: 84.4%, R0: 100%
Kawamura [68]	Retrospective	2018	Japan	U-EMR	10, 13 or 33 mm rotatable snares	Pulse-cut slow (20 W) Forced coag. (2)	Any size	64	C: 9 (14) AC: 12 (19) TC: 12 (19) DC: 7 (11) SC: 17 (27) R: 7 (11)	81%	54%		Bleeding: 3 (5) Perforation: 1 (2%)	-
Siau [67]	Single-centre RCT	2018	UK	U-EMR	25 mm snare, other types	Left colon: 30 W E 2 Right colon: 20 W E 2	>10 mm	97	C: 13 (13.4) AC: 7 (7.2) TC: 12 (12.4) DC: 1 (1.0) SC: 24 (24.7) R: 40 (41.2)	82.9%	-	8/59 underwent follow-up	4 (4.1)	-
Chaves [69]	Retrospective	2018	Brazil	U-EMR	15–25 mm multifilament snare	Endocut (E 3, I 6, L 1)	>10 mm	16	C: 1 (6.3) AC: 10 (62.4) TC: 4 (25) DC: 1 (6.3)	8 (50.0%)	-	-	0	-
Yamashina [70]	Multicentre RCT	2019	Japan	U-EMR	-	Endocut or pulse-cut mode	10–20 mm	108	C: 16 (15) AC: 21 (19) TC: 29 (27) DC: 11 (10) SC: 23 (21) R: 8 (7.4)	89%	69%		Bleeding: 3 (2.8)	Conventional EMR (*en bloc*: 75%, R0: 50%)
Chien [71]	Retrospective	2019	Taiwan	U-EMR	-	-	>10	121	Right C: 94 (52.5) Left C: 77 (43.0)	141 (82.5)	-	-	Bleeding: 11 (6.5) Perforation: 4 (2.4)	Conventional EMR (*en bloc*: 87.6%)
Zhang [72]	Multicentre RCT	2020	China	U-EMR	Round snare	Endo cut Q (E 4, I 6, L 1) Forced coag. (E 2)	4–9 mm	71	AC: 13 (18.3) TC: 21 (29.6) (29.6) DC: 6 (8.4) SC: 22 (31.0) R: 9 (12.7)	94.4%	-	-	Bleeding: 1 (1.5)	CSP *En bloc:* 91.5%
Yen [73]	Single-centre RCT	2020	USA	U-EMR	6–9 mm: 9 mm dedicated cold snare >10 mm: 15 mm firm monofilament hot snare	Endocut Q, (E 3, I 3, L 1)	6–20 mm >20 mm	248	C: 25 (10.1) AC: 67 (27.0) TC: 110 (44.4) DC: 15 (6.1) SC: 21 (8.5) R: 10 (4.0)	Overall: 89.9% 6–9 mm: 97.2% 10–19 mm: 84.6% >20 mm: 25%	-	-	Bleeding: 10 (4.0)	6–9 mm: CSP >10 mm: conventional EMR Overall R0: 90.2%
Barclay [74]	Retrospective	2020	USA	U-EMR	Stiff rounded 25–33 mm snares	Endocut Q (E 2, I 4, L 1)	>20 mm	264	C: 87 (33) AC: 64 (24) TC: 41 816) DC: 16 (6) R: 24 (9)	28%	-	10/174 underwent follow-up	Bleeding: 43 (16.3)	-
Nagl [8]	Single-centre RCT	2021	Germany	U-EMR	15–25 mm snares	Endocut Q (E 2) Forced coag.	20–40 mm	81	C: 20 (24.7) AC: 28 (34.6) TC: 7 (8.6) DC: 5 (6.2) SC:6 (7.4) R:0	33.3%	32.1%	15.1%	Bleeding: 19 (23.5%) intra, 1 (1.2) post-procedural	Conventional EMR (*en bloc*: 18.4%, R0 15.8%, recurrence 24.6%)
Inoue [75]	Retrospective	2021	Japan	U-EMR	15–30 mm snare	-	20–30 mm	125	Right C: 99 (79.2) Left C: 23 (18.4%) Rectum: 3 (2.4%)	61%	45%	2/97 underwent follow-up	Bleeding: 5 (4%) Perforation: 1 (0.8)	ESD (*en bloc*: 99%, R0: 86%, recurrence: 0)
Nogueira [9]	Prospective	2021	Brazil	U-EMR	Stiff rounded snares	Endocut (E 4)	>5 mm	51	Right C: 38 (74.5) Left C: 10 (19.6) R: 3 (5.8)	52.9%	-	-	Bleeding: 6 (11.8)	-
Iwagami [76]	Retrospective	2022	Japan	U-EMR	Stiff rounded 10–25 mm snares	Endocut Q (E3/2) Forced coag. (3/4.5)	>20 mm	52	AC: 28 (54) TC: 12 (23) DC: 3 (6) SC: 5 (9) R:4 (8)	75%	73%	-	Bleeding: 1 (1.9) Perforation: 1 (1.9)	-
Okada § [77]	Retrospective	2022	Japan	U-EMR	Rounded 15-mm snare	Pure-cut	>20 mm	11	Right C: 10 (91%) Left C: 1 (9%)	91%	91%	-	0	-
Uchima [78]	Retrospective	2023	Spain	U-EMR Cap suction	Stiff rounded 10–25 mm snares	Endocut Q (E 2) Pulse-cut slow (E 2)	20 mm (15–30)	83	Appendiceal orifice: 11 (13.3) Ileo-caecal valve: 8 (9.6) C: 17 (20.5) AC: 10 (12) TC: 25 (30.1) DC: 2 (2.4) SC: 8 (8.6) R: 2 (2.4)	54.2%	-	-	Bleeding: 9 (10.8)	-
Kim [79]	Single-centre prospective	2023	Republic of Korea	U-EMR	Hexagonal snare	Endocut Q (E 1, I 3, L 3)	10–20 mm	47	C: 10 (21.3) AC: 10 (21.3) TC: 12 (25.5) DC: 6 (12.8) SC: 7 (14.9) R: (4.3)	97.9%	80.9%	-	Bleeding: 6 (12.8)	-
Okimoto [80]	Single-centre RCT	2023	Japan	U-EMR	Rounded snare	-	21–30 mm	11	C: 3 (27) AC: 2 (18) TC: 2 (18) DC: 0 SC: 3 (27) R: 1 (9.1)	82%	36%	1/8 underwent follow-up	0	ESD (*en bloc*: 100%, R0: 100%, recurrence: 0/10)
Ashizawa ^ [81]	Retrospective	2023	Japan	U-EMR	Stiff rounded 10–25 mm snares	Endocut Q (E 3, I 2, L 2)	>10 mm	25	C: 6 (24) AC: 8 (32) TC: 4 (16) DC: 3 (12) SC: 4 (16) R: 0	80%	72%	-	0	-
Rodríguez Sánchez [10]	Multicentre RCT	2023	Spain	U-EMR	Wide range of snare types, endoscopist’s discretion	Endocut Q (E 2, I 6, L 1)	>10 mm	149	Right C: 71 (47.7) Left C: 78 (52.3)	31.5%	27.5%	9.5%	Bleeding: 31 (20.8) Perforations: 4 (2.7) Others: 7 (4.7)	Conventional EMR: *en bloc*: 28.4%, R0: 24.1%, recurrence: 11.7%
Lenz [82]	Multicentre RCT	2023	Brazil	U-EMR	Stiff rounded 13–25 mm snares	Endocut Q (E 3, I 6, L 1)	10–40 mm	61	Right C: 41 (67.2) Left C: 20 (32.8)	60.7%	-	2%	2 (3.3%)	Conventional EMR: *en bloc*: 54.2%, recurrence: 8 (15%)
Nagata [58]	Retrospective	2017	Japan	U-ESD	Waterjet-not-assisted knife	Endocut I Swift coag. (3)	22.5 (17.8–25.3)	26	C: 5 (19.2) AC: 4 (15.4) TC: 7 (26.9) DC: 0 (0) SC: 4 (15.4) R: 6 (23.1)	100%	-	-	1 (83.8)	-
Yoshii [83]	Retrospective	2018	Japan	U-ESD	Bipolar needle knife with water-jet function	Endocut I (E 3, I 3, L 3) Swift-coag. (2) Dry-cut (3)	>20 mm	40	C: 4 (10) AC: 7 (17.5) TC: 9 (22.5) DC: 4 (10) SC: 7 (17.5) R: 9 (22.5)	100%	87.5% °	-	0	-
Cecinato [84]	Retrospective	2022	Italy	U-ESD	Waterjet system-assisted or not assisted knife	Endocut Q (E 3) Swift coag. (3) Spray coag. (4)	44.5 (±17.8)	22	Right C: 10 (45.5) Left C: 5 (22.7) R: 7 (31.8)	100%	100% °	0%	Other: 1 (5%)	Hybrid ESD: *en bloc*: 59.5%, R0:54.5%, recurrence: 2.7%
Koyama [85]	Retrospective	2023	Japan	U-ESD	Waterjet-not-assisted knife	Endocut I (E 2, I 2, L 2) Swift coag. (3) Forced coag. (3)	22 (18–27)	80	Right C: 54 (68) Left C: 20 (25) Rectum: 6 (7)	98.7%	98.7 °	-	2 (2.5%)	Conventional-ESD: *en bloc*: 99.2, R0: 94.4%
Shigeta [86]	Case report	2023	Japan	U-EFTR	Full thickness resection device system	-	-	1	AC: 1	100%	100%	-	0	

Abbreviations: ESU, electrosurgical unit; U-EMR, underwater endoscopic mucosal resection; EMR, endoscopic mucosal resection; CSP, cold snare polypectomy; ESD, endoscopic submucosal dissection; C, caecum; AC, ascending colon; TC, transverse colon; DC, descending colon; SC, sigmoid colon; R, rectum; E, effect, I, interval, L, length; U-EFTR, underwater endoscopic full-thickness resection. R0: *en bloc* with uninvolved margins. * Not same R0 as above. ° R0 intended as curative resection. ^ Gel-immersion EMR. § Progressive polyp contraction.

## Abbreviations

Gastrointestinal (GI); cold snare polypectomy (CSP); endoscopic mucosal resection (EMR); European Society of Gastrointestinal Endoscopy (ESGE); endoscopic submucosal dissection (ESD); full-thickness resection (FTR); peroral endoscopic myotomy (POEM); peroral endoscopic septotomy (POES); underwater EMR (U-EMR); underwater (U); Society of American Gastrointestinal and Endoscopic Surgeons (SAGES); ESU, electrosurgical unit; GEJ, gastroesophageal junction; prophylactic underwater coagulation peroral endoscopic myotomy (PUC-POEM); superficial non-ampullary duodenal epithelial tumours (SNADET); randomized controlled trial (RCT); caecum (C); ascending colon (AC); transverse colon (TC); descending colon (DC); sigmoid colon (SC); rectum (R); laparoscopic endoscopic co-operative surgery (LECS).
